# A Theoretical Study of the C–X Bond Cleavage Mediated by Cob(II)Aloxime

**DOI:** 10.3390/molecules27217283

**Published:** 2022-10-26

**Authors:** Luis E. Seijas, Cesar H. Zambrano, Vladimir Rodríguez, Jorge Alí-Torres, Luis Rincón, F. Javier Torres

**Affiliations:** 1 Grupo de Química Computacional y Teórica (QCT-UR), Facultad de Ciencias Naturales, Universidad del Rosario, Bogotá 111221, Colombia; 2 Grupo de Química Computacional y Teórica (QCT-USFQ), Departamento de Ingeniería Química, Universidad San Francisco de Quito, Diego de Robles y Vía Interoceánica, Quito 17-1200-841, Ecuador; 3 Departamento de Matemática, Universidad San Francisco de Quito, Diego de Robles y Vía Interoceánica, Quito 17-1200-841, Ecuador; 4 Departamento de Química, Universidad Nacional de Colombia, Av. Cra. 30 #45-03, Bogotá 111321, Colombia

**Keywords:** reaction force, atoms in molecules, source function, cobaloxime, carbon–halogen cleavage

## Abstract

The C–X bond cleavage in different methyl halides (CH_3_X; X = Cl, Br, I) mediated by 5,6-dimethylbenzimidazole-bis(dimethylglyoximate)cobalt(II) (Co^II^Cbx) was theoretically investigated in the present work. An S_N_2-like mechanism was considered to simulate the chemical process where the cobalt atom acts as the nucleophile and the halogen as the leaving group. The reaction path was computed by means of the intrinsic reaction coordinate method and analyzed in detail through the reaction force formalism, the quantum theory of atoms in molecules (QTAIM), and the calculation of one-electron density derived quantities, such as the source function (SF) and the spin density. A thorough comparison of the results with those obtained in the same reaction occurring in presence of 5,6-dimethylbenzimidazole-bis(dimethylglyoximate)cobalt(I) (Co^I^Cbx) was conducted to reveal the main differences between the two cases. The reactions mediated by Co^II^Cbx were observed to be endothermic and possess higher activation energies in contrast to the reactions where the Co^I^Cbx complex is present. The latter was supported by the reaction force results, which suggest a relationship between the activation energy and the ionization potentials of the different nucleophiles present in the cleavage reaction. Moreover, the SF results indicates that the lower axial ligand (i.e., 5,6-dimethylbenzimidazole) exclusively participates on the first stage of the reaction mediated by the Co^II^Cbx complex, while for the Co^I^Cbx case, it appears to have an important role along the whole process. Finally, the QTAIM charge analysis indicates that oxidation of the cobalt atom occurs in both cases; at the same time, it suggests the formation of an uncommon two-center one-electron bond in the Co^II^Cbx case. The latter was confirmed by means of electron localization calculations, which resulted in a larger electron count at the Co–C interatomic region for the Co^I^Cbx case upon comparison with its Co^II^Cbx counterpart.

## 1. Introduction

Organohalides, also known as halocarbons, are a family of organic compounds characterized by the presence of at least one carbon–halogen bond (C–X; X = F, Cl, Br, I) in their molecular structure [1]. Although it has been reported that a significant number of these compounds occur naturally as a product of different biochemical as well as geological processes [2,3,4], the most known and common members of this family of compounds have an anthropogenic origin. As a matter of fact, due to their outstanding physicochemical properties, organohalides have been designed and synthetized to be used in several applications, such as solvents, refrigerants, polymers, foams, elastomers, flame retardants, intermediates in synthetic organic chemistry, and others [5,6]. The great spectrum of uses of organohalides has driven their indiscriminate industrial production, which in turn has resulted in their diffusion into the atmosphere, water sources, and soil [6]. It is important to point out that the worldwide spread of organohalides has not been exclusively attributed to the emissions during their production, since the reckless use, the inadequate disposal, and most importantly, the lack of waste treatments have been determined as significant sources of environmental contamination [7]. In addition to the environmental concerns, organohalides have been observed to bioaccumulate in macro-organisms, and they have been branded as probable human carcinogens [7,8,9]. The latter issues have led the inclusion of several members of the halocarbon family in the list of hazardous persistent organic pollutants (POPs) under the United Nations Stockholm Convention [10]. Furthermore, several countries have proposed and approved laws to regulate or even restrict future production, use and disposal of organohalides [10].

**Scheme 1:**
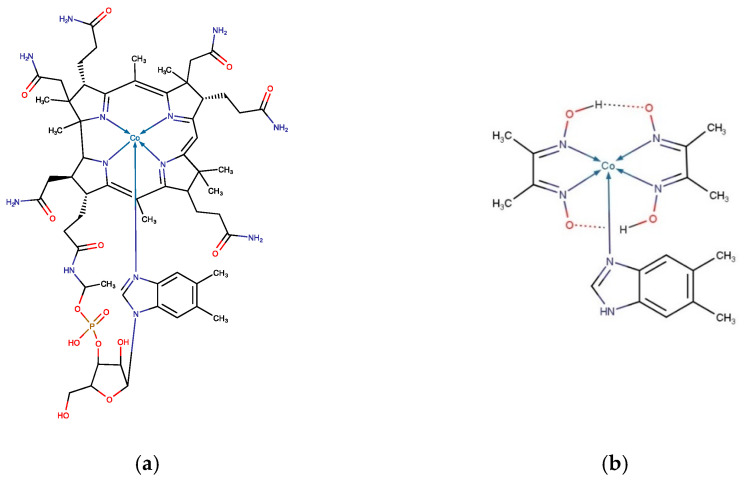
Schematic representations of compounds (**a**) cobalamin and (**b**) 5,6-dimethylbenzimidazole-bis(dimethylglyoximate)cobalt(II) (cob[II]aloxime or Co^II^Cbx).

On such panorama, intense research has been conducted to develop viable organohalide degradation methods. It is well known that organohalides are resistant to traditional physical and chemical treatments; therefore, the proposed methods for the degradation of these very recalcitrant compounds consider either high-energy electrochemical processes [11,12] or photocatalytic oxidation [13]. Although the latter have proved to be highly effective in the elimination of organohalides, several drawbacks avoid their application at an industrial level [7,10]. In this context, the biodegradation of organohalides emerges as a very promising approach since several anaerobic bacteria able to reductively dehalogenate aliphatic and aromatic organohalides have been identify and isolated [14]. To better understand the details of this biological process, anaerobic biotransformation experiments [14,15] have been also conducted, where it has been shown that vitamin B_12_ (i.e., cyanocob[N]alamin, Cbl, a corrinoid macromolecular complex containing a central cobalt atom bearing different oxidation states: Co^I^, Co^II^, and Co^III^; see Scheme 1a) plays a fundamental role in the reductive dehalogenation process of organohalides. For instance, Becker and Freedman showed that the biodegradation of chloroform by means of a methanogenic culture grown on dichloromethane as the sole carbon and energy source, is significantly enhanced upon the addition of supplemental Cbl. In a more recent work, Guerrero-Barajas and Field [16] showed that the inclusion of Cbl biosynthetic precursors have the same effect in the biodegradation of carbon tetrachloride [17]. On the other hand, biomimetic essays carried out by considering different electron donors and Cbl as the electron shuttle [18,19,20,21,22,23] have shown that Co^III^ is not active, and Cbl forms containing reduced forms of cobalt are therefore required for the reaction to occur [24]. Nonetheless, precise information on the exact oxidation state of the reduced cobalt atom has been elusive. In these regards, it is important to point out that reduction from Co^III^ to Co^II^ is thermodynamical favorable with a reduction potential of 0.20 eV, whereas the subsequent Co^II^ to Co^I^ reduction is unfavorable, as inferred from its relatively high negative reduction potential of −0.61eV. Therefore, the specific oxidation state of Co[N]Cbl in biomimetic experiments has been observed to highly depend on the electron donor included in the reactive system [22]. 

**Scheme 2:**
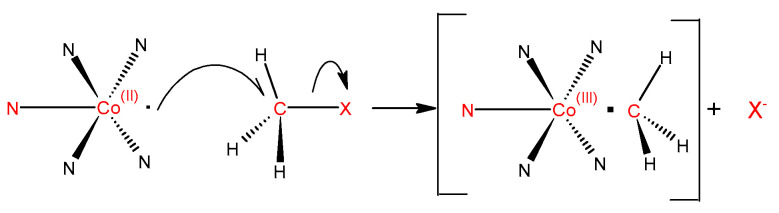
Schematic representation of reaction between Co^II^Cbx and methyl halide, where the three atoms that are relevant for the discussion of the reaction mechanism are emphasized in red color.

In the latter context, the Co^I^Cbl system, also known as “superreduced” Vitamin B_12_[25] has been determined to act as an extremely strong reductant agent [26], capable of efficiently attacking the electrophile carbon atoms belonging to C–X bonds in organohalides [24]. Accordingly, this system has been extensively investigated by different experimental as well as theoretical approaches [27,28,29,30,31,32,33,34,35]. In contrast, the Co^II^Cbl system, which contains the more common Co^II^ ion (i.e., a *d^7^* metal center), has been less explored in the literature. As reported by Lewis et al. [22], Co^II^Cbl has been observed to occur in carbon tetrachloride degradation biomimetic essays where dithiothreitol or cysteine are employed as electron donors. Moreover, its presence in the reactive system has suggested to promote reaction pathways characterized by the formation of radicals and the consequent one-electron reduction steps [36]. Since the aforementioned dehalogenation pathways are not well understood, the present study aims to shed light on the C–X (X = Cl, Br, I) bond cleavage mediated by Co^II^-based compounds. In order to avoid excessive computational costs, the cob[II]aloxime, Co^II^Cbx (Scheme 1b), complex (i.e., ~50 atoms/molecule) is considered as a substitute for the Co^II^Cbl system (i.e., ~200 atoms/molecule). The latter is justified on the basis of two considerations: (i) Schrauzer and Deutsch have experimentally shown that reduced forms of Co[N]Cbx are able to act as electron shuttles in the reductive dehalogenation process of organohalides [37,38,39], and (ii) a previous computational study by some of the authors of this study has demonstrated that the Co^I^Cbx can be used as a reliable theoretical model of Co^I^Cbl [35]. As in our previous work, the C–X bond cleavage mediated by Co^II^Cbx is considered to occur via a S_N_2 mechanism, where the reduced cobalt atom acts as the nucleophile and the halogen as the leaving group as illustrated in Scheme 2. The results obtained for the latter chemical process are herein thoroughly analyzed by means of the reaction force formalism, the quantum theory of atoms in molecules, and the computation of one-electron density derived quantities, such as: the source function and the spin density; and emphasizing the main differences in the reaction occurring in the presence of Co^I^Cbx as previously reported, as well as re-considered in the present work. Finally, a further analysis of the electron localization computed through information-theory based approaches is also presented to characterize the electronic features of the products of the reaction.

## 2. Models and Methods 

The methodology thoroughly described in Reference [35] was adopted to construct the models in the present study. It is necessary to mention that in Reference [35], the Co[N]Cbx was proven to be a reliable theoretical model describing the reductive dehalogenation of methyl halides. For the sake of completeness, this procedure will be briefly summarized here. As a starting point, the methylated Co^II^Cbx (i.e., the product of the reaction in Scheme 2) was fully optimized at the unrestricted uωB97XD/6-311++G(d,p) level of theory as the doublet nature of the system has to be considered in the calculations. Default threshold values for the optimization processes as implemented in the GAUSSIAN16 suit of programs were considered [40]. Upon obtaining the equilibrium geometry of the CH_3_Co^II^Cbx compound, a halide X^−^ anion (i.e., X = Cl, Br, I) was added to the structure close to the carbon of the CH_3_ moiety, which was also modified to resemble a *sp2* geometry and thus obtain an approximate transition-state structure (Scheme 2). It is worth mentioning that no pseudo-potential forms were employed to describe the I^−^ anion; instead, the all-electron 6-311(d,p) basis set was adopted. The approximated structures were employed as the starting point for a further transition-state optimization process. Moreover, a subsequent vibrational analysis was conducted to confirm that each computed TS structure corresponded to a saddle point on the potential energy surface (PES). Solvent effects were included in all the calculations via the Cramer and Truhlar’s SMD solvation model [41,42] with a dielectric constant value of 78.3553, corresponding to water. Although results for the CH_3_Cl dehalogenation mediated by Co^I^Cbx are reported in Reference [35], some calculations for this reaction system were repeated here in order to have a common line of comparison.

In a further step of the study, the reaction coordinate for the C–X bond cleavage process, mediated by Co^II^Cbx was obtained by means of the intrinsic reaction coordinate (IRC) algorithm [43,44,45]. Then, the IRC results were analyzed through the reaction force (RF) formalism, which has been proposed as an efficient method to unveil the more important details of a chemical reaction. A complete discussion on the RF formalism can be reviewed in References. [46,47,48]; here, it is only reminded that the RF (Equation (1)) is defined as the negative of the derivative of potential energy, E(ξ) with respect to the scaled intrinsic reaction coordinate (ξ):

(1) $$F\left(\xi \right)=-\frac{dE\left(\xi \right)}{d\xi }$$

The maximum on the IRC, which corresponds with $F\left(\xi_{TS}\right)=0$, defines a partition of ξ in two segments. The first one corresponds to an endothermic process where the system goes from the reactants state to the transition state (i.e., $\xi_{R}$ to $\xi_{TS}$). The second segment is an exothermic process related to the relaxation from the transition state to the products (i.e., $\xi_{TS}$ to $\xi_{R}$). Hereinafter, the two different IRC sections are referred to as “segment of reactants” (SR) and “segment of products” (SP), respectively. Within the RF formalism, each of these segments would contain *n* and *m* critical points on F(ξ) that can be readily employed as integral limits for the calculation of the so-called reaction works (in the analogy with the mechanical definition, Equation (2)) [48,49]:

(2) $$w_{i}^{j}=-\int_{\xi_{i}^{j}}^{\xi_{i+1}^{j}}F\left(\xi \right)d\xi ,\;i=1,\;2,\;\ldots ;\;j=SR,\;SP$$

In fully concerted reactions, one critical point is found at each segment (*n* = *m* = 1) allowing the definition of four reaction works. The two belonging to SR ($w_{i}^{SR}$) are positive, and they represent the resistance to activation, while the other works are associated with SP ($w_{i}^{SP}$) are negative and represent the relaxation process. Considering the latter, the activation energy and the reaction energy can be obtained by Equations (3) and (4), respectively.

(3) $$\Delta E^{\neq }=\overset{n+1}{\underset{i}{\sum }}w_{i}^{SR}$$

(4) $$\Delta E^{0}=\Delta E^{\neq }+\overset{m+1}{\underset{i}{\sum }}w_{i}^{SP}$$

For the reductive dehalogenation of CH_3_–Cl mediated by the different Co-based compounds (i.e., Co^I^Cbx and Co^II^Cbx), the critical points found in the RF were also examined using the Quantum Theory of Atoms in Molecules (QTAIM) [50]. In this theory, the electron density embodies a natural partition scheme allowing the separation of regions, Ω, identified as atoms in molecules. The boundary of each atom is defined by a zero-flux surface in the gradient vector field of the electron density. An important quantity derived from the electron density is the Laplacian, $\nabla^{2}\rho$, that can be used to distinguish between attractive ($\nabla^{2}\rho$ < 0, concentration of electron density in the reference point, relative to the surroundings) and repulsive interactions ($\nabla^{2}\rho$ > 0, depletion of electron density). This quantity can be written as the sum of contributions along the three principal axes of maximum variation, that is, the eigenvalues ($\lambda_{i}$) of the Hessian matrix: $\nabla^{2}\rho =\lambda_{1}+\lambda_{2}+\lambda_{3}$. Moreover, the algebraic sum of the signs of $\lambda_{i}$ (σ) together with the number of the non-zero curvatures, also known as rank (*ω*), allow the classification of the electron density critical points. In this context, given *ω* = 3 (i.e., stable critical points), there are four different types of critical points: (i) nuclear critical points (3, −3), (ii) bond critical points (3, −1), (iii) ring critical points (3, +1), and (iv) cage critical points (3, +3). Apart of the characterization of the system in terms of the different kind of critical points, the QTAIM atomic charges were also calculated in the present study. Moreover, for the case of reaction mediated by Co^II^Cbx, the spin population was computed to obtain information about the spin polarization in this particular case.

The evolution of the density critical points along the reaction path was investigated using the source function (SF) introduced by Bader and Gatti in 1998 [51]. This quantity shows that the electron density at a given point in the space is determined by a Green’s function. This means that the electron density at **r** is defined as the sum of contributions of different local sources $LS\left(r,r^{\prime }\right)$, operating at all the other points of the space [52,53]. Therefore, the electron density can be written as in Equation (5)

(5) $$\rho \left(r\right)=\int LS\left(r,r^{\prime }\right)dr^{\prime }$$

where the local source, given by $LS\left(r,r^{\prime }\right)=-{\left(4\pi \left|r-r^{\prime }\right|\right)}^{-1}\cdot \nabla^{2}\rho \left(r^{\prime }\right)$, represents how the Laplacian of the electron density at the position $r^{\prime }$ affects the electron density at a different position r [25]. Consequently, the integral of $LS\left(r,r^{\prime }\right)$ over an atomic QTAIM basin [50] provides information related to the contributions of the basin Ω to the electron density at r; therefore, ρ(r) can be written as a sum of atomic source contributions (Equations (6) and (7)):

(6) $$\rho \left(r\right)=S\left(r,\Omega \right)+\underset{\Omega^{\prime }\neq \Omega }{\sum }S\left(r,\;\Omega^{\prime }\right)$$

where,

(7) $$S\left(r,\Omega \right)=\int_{\Omega }LS\left(r,r^{\prime }\right)dr^{\prime }$$

is defined as the source function. 

According to Equation (6) ρ(r) belonging to a basin Ω can be envisaged as a self-contribution S(r,Ω), and the sum of contributions from the other atoms in the molecule $S\left(r,\Omega^{\prime }\right)$. Furthermore, by employing the local expression of the virial theorem $\nabla^{2}\rho \left(r\right)=4\left[2G\left(r\right)+V\left(r\right)\right]$, Ref. [50] $LS\left(r,r^{\prime }\right)$ can be expressed as in Equation (8):

(8) $$LS\left(r,r^{\prime }\right)=-\frac{1}{\pi }\times \frac{2G\left(r^{\prime }\right)+V\left(r^{\prime }\right)}{\left|r-r^{\prime }\right|}$$

where $G\left(r^{\prime }\right)$ is the positively defined kinetic energy density, and $V\left(r^{\prime }\right)$ is the electronic potential energy density. According to Equation (8), molecular regions, where the potential energy density $V\left(r^{\prime }\right)$ overcomes the kinetic energy density $G\left(r^{\prime }\right)$, act as positive sources of the electron density ρ(r) and *vice versa.* In this context, it is obvious that the SF S(r,Ω), can take positive or negative values. Moreover, for an atom in a molecule, the local positive sources are usually the predominant terms in the definition of the electron density associated with a bond critical point (BCP). However, the opposite may also occur in specific circumstances such as transition states [53]. In this work, the source function values will be also expressed as the percentage contribution to the electron density at **r**, as show in Equation (9).

(9) $$S\%\left(r,\Omega \right)=\left[\frac{S\left(r,\Omega \right)}{\rho \left(r\right)}\right]\times 100$$

It is important to point out that S(r,Ω) (Equation (7)) and S%(r,Ω) (Equation (9)) have a different meaning. S(r,Ω) is related to the nature and strength of the associated interaction between the atoms intervening into the BCP and the relative contribution from Ω to $\rho_{BCP}$, whereas S%(r,Ω) express the percentage of sharing from Ω to $\rho_{BCP}$. An important property of S%(r,Ω) is that it reflects the delocalization character of a given interaction [51]. Both, S(r,Ω) and S%(r,Ω) will be used in the present work to analyze the bond evolution along the RF critical points. The wavefunctions obtained with the GAUSSIAN16 suit of programs were used as input for the Multiwfn package [54].

Finally, the electron localization on the Co–C bond during the reductive dehalogenation of CH_3_–Cl mediated by Co^I^Cbx and Co^II^Cbx was computed using the information contained in the same spin conditional pair density extracted by means of the Kullback–Leibler divergence (KLD), as is show in Equation (10) [55,56,57,58]:

(10) $$D_{KL,XC}^{\sigma }\left(r_{1}\right)=\int dr_{2}\rho_{cond}^{\sigma \sigma }\left(r_{2}|r_{1}\right)\log_{2}\frac{\rho_{cond}^{\sigma \sigma }\left(r_{2}|r_{1}\right)}{\sigma^{\sigma }\left(r_{2}\right)},$$

where ρcondσσ(r2|r1)=γcondσσ(r2|r1)Nσ−1, represents the same spin conditional pair density and σσ(r2)=ρσ(r2)Nσ is the σ-spin shape function. The interested reader is referred to Ref. [58] for detailed information on the theoretical aspects of this electron localization method. Here, it is only reminded that, under the Kullback–Leibler Divergence interpretation, $D_{KL,XC}^{\sigma }$, can be considered as a measure of the information content in the exchange-correlation hole density, measured as bits times electron (bte); thus, Equation (10) can be readily used to define a descriptor of the electron localization in real space (Equation (11)),

(11) $$\chi_{XC}^{\sigma }\left(r_{1}\right)=(N^{\sigma }-1)\;D_{KL,XC}^{\sigma }\left(r_{1}\right)f_{cut}\left(r_{1}\right),$$

where $f_{cut}\left(r\right)$, is a cut-off function that goes smoothly to zero for negligible density values [57].

As in the case of the SF, the $\chi_{XC}^{\sigma }$ was computed from wavefunctions obtained with the GAUSSIAN16 suit of programs by employing our in-house developed C++/CUDA program, called KLD and compiled with NVIDIA CUDA. A Tesla P-100 GPU was employed for the calculations, which were divided into four stream flows, each one of them containing about the same number of points to be evaluated [59]. The KLD code is freely available upon request. The tridimensional graphical visualization of the results was produced using the VMD program [60].

## 3. Results and Discussion

### 3.1. IRC and Reaction Force (RF)

**Figure 1:**
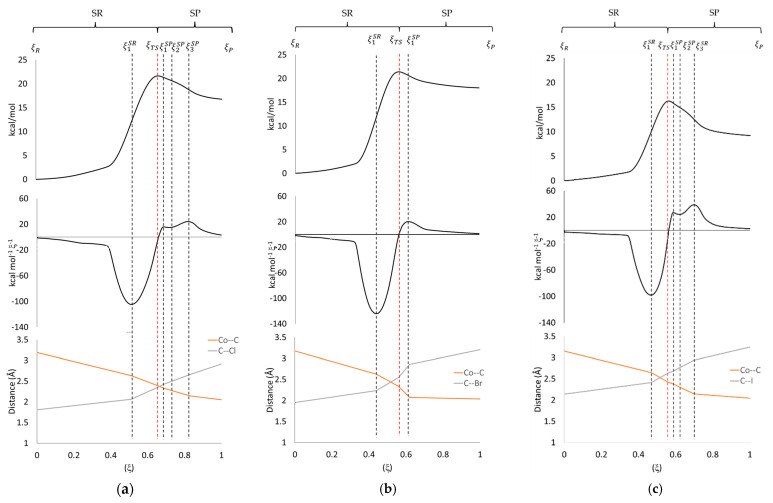
IRCs and reaction force curves, calculated at uωB97-XD/6–311++G(d,p) level, for (**a**) Cl, (**b**) Br and (**c**) I as the leaving group in reaction. The dashed lines show the position of critical points in the normalized reaction coordinate.

**Figure 2:**
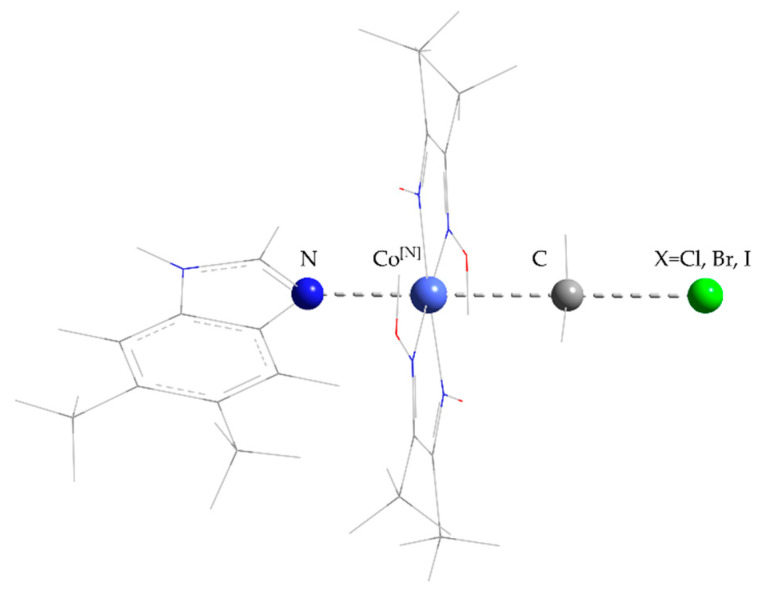
Graphical representation of the transition state Co[N]Cbx···CH_3_···X (X = Cl, Br, I) emphasizing the relevant bonds.

Figure 1 shows the normalized IRC, the RF profiles, and the changes in the bond distances that are relevant for the reaction (i.e., Co–C and C–X; X = Cl, Br, and I, as shown in Figure 2). For each reaction, the reactant corresponds to the fully optimized non-covalent complex formed between Co^II^Cbx and the respective methyl halide. Inspection of Figure 1 reveals the endothermic nature of the reactions considered, indicating that the methyl halide dehalogenation mediated by Co^II^Cbx is energetically less favorable than its counterpart mediated by Co^I^Cbx (Table 1). A further examination of the RF profiles reveals that only the reaction between the CH_3_Br and Co^II^Cbx presents one minimum and one maximum in the RF profile (Figure 1b). For the other two reactions, two maximums at the segment of products (SP) were observed (Figure 1a,c). These results may be related with changes in the slope energy curve along the SP, due to geometric, or electronic factors that occur during the C–Cl and C–I bonds cleavage. As a matter of fact, in these cases, the rate of the Co–C bond distance shortening as well as C–X elongation slightly varies from TS ($\xi_{TS}$) to the products ($\xi_{P})$. However, this behavior does not affect the estimation of the activation energies (see below). Regarding the latter quantities, data reported in Table 1 show that the activation energies follow the I < Br < Cl trend in agreement with the leaving group ability, a fact that was also observed for the case of the methyl halide dehalogenation mediated by Co^I^Cbx, as reported in Table 1 (data between parentheses). As determined by the RF formalism, two works (i.e., $w_{1}^{SR}$ and, $w_{2}^{SR}$) can be readily defined in the SR segment of the three reactions (see Equation (2)). The first one is related principally to the geometrical rearrangements occurring when the reaction goes from $\xi_{R}$ to $\xi_{1}^{SR}$, while the second one is mainly associated with the electronic reorganization in the path from $\xi_{1}^{SR}$ to $\xi_{TS}$. According to Equation (3) (Models and Methods Section) the activation energy can be expressed as the sum of $w_{1}^{SR}$ and, $w_{2}^{SR}$; therefore, each work can be seen as a fraction of the activation energy, as follows: $w_{1}^{SR}$ ≈ 0.52ΔE^≠^ and $w_{2}^{SR}$ ≈ 0.48ΔE^≠^ for the C–Cl cleavage, $w_{1}^{SR}$ ≈ 0.55ΔE^≠^ and $w_{2}^{SR}$ ≈ 0.45ΔE^≠^ for the C–Br cleavage, and $w_{1}^{SR}$ ≈ 0.58ΔE^≠^ and $w_{2}^{SR}$ ≈ 0.42ΔE^≠^ for C–I case. The latter results indicate that the geometrical rearrangements have a greater contribution to the activation energy in the first step of the C–X cleavage. 

A similar analysis can be carried out in the segments of the products (SP), where the forces are positive (i.e., energy release), and they could also be related with electronic and geometrical rearrangements of the system in the path from $\xi_{TS}$ to $\xi_{P}$. For the C–Br cleavage, two works were defined: $w_{1}^{SP}$ is related to the electronic changes in the path from $\xi_{TS}$ to $\xi_{1}^{SP}$, and $w_{2}^{SP}$ is associated with geometrical rearrangements in the path from $\xi_{1}^{SP}$ to $\xi_{P}$. An examination of Table 1 shows that for this case, $\left|w_{1}^{SP}\right|<\left|w_{2}^{SP}\right|$; therefore, the system gains greater stabilization due to the geometrical rearrangements that occur when the products are formed. In the cases of the C–Cl and C–I the additional maximum observed in the SP have led to the definition of four works $w_{1}^{SP}$, $w_{2}^{SP}$, $w_{3}^{SP}$ and $w_{4}^{SP}$, related to the changes observed in the paths $\xi_{TS}$ to $\xi_{1}^{SP}$, $\xi_{1}^{SP}$ to $\xi_{2}^{SP}$, $\xi_{2}^{SP}$ to $\xi_{3}^{SP}$, and $\xi_{3}^{SP}$ to $\xi_{P}$, respectively. Of them, $w_{2}^{SP}$ and $w_{4}^{SP}$ could be associated with the geometrical rearrangement of the products, while $w_{1}^{SP}$ and $w_{3}^{SP}$ could be associated with electronic changes. Moreover, it is observed that $\left|w_{1}^{SP}+w_{3}^{SP}\right|<\left|w_{2}^{SP}+w_{4}^{SP}\right|$ in both cases. 

**Scheme 3:**
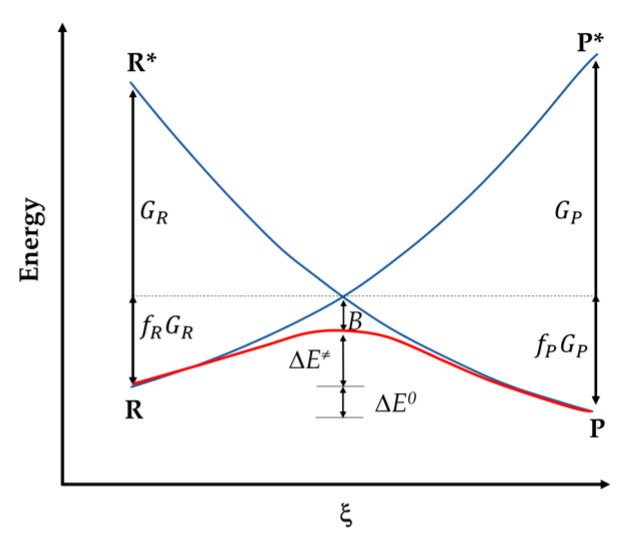
Schematic valence bond state correlation diagram (VBSCD) showing the key factors that affect the barrier.

In order to avoid presenting a plethora of comparisons, a more straightforward discussion is presented; thus, after this point, the discussion will focus on the C–Cl cleavage mediated by both Co^I^Cbx and Co^II^Cbx complexes. From Table 1, it is observed that methyl dehalogenations occurring in the presence of Co^II^Cbx have activation energies greater than those reported for the same reactions mediated by Co^I^Cbx. With the purpose of unveiling the main reasons for this, an alternative interpretation of the RF, previously reported by some of the authors of the present work, is employed [61]. This RF picture requires a briefly description of the valence bond state correlation diagram (VBSCD) depicted in Scheme 3, where it is illustrated that the energy required to promote the system from R to its excited state R* is denoted by $G_{R}$, and B is the quantum mechanical resonance energy that separates the crossing of the diabatic curves and the maxima of the adiabatic potential energy. The activation energy of a given reaction, $\Delta E^{\neq }$, can be expressed as: $\Delta E^{\neq }=f_{R}G_{R}-B$, where $f_{R}$ is the fraction (i.e., defined in the ]0,1] range) of $G_{R}$ that contributes to the activation energy. Written in the latter form, $\Delta E^{\neq }$ can be envisaged as the interplay between a destabilizing term ($f_{R}G_{R}$) and a stabilizing one (B). Moreover, as stated in Reference. [61], the ${\left[B/\left(f_{R}G_{R}\right)\right]}^{2/3}$ can be approximated to $w_{2}^{SR}/w_{1}^{SR}$; thus, the activation energy can be expressed as $\Delta E^{\neq }\approx f_{R}G_{R}\left[1-{\left(w_{2}^{SR}/w_{1}^{SR}\right)}^{3/2}\right]$, which, in the present study, is used to estimate the $f_{R}G_{R}$ term. It is important to point out that the reaction occurring in the presence of Co^I^Cbx was recalculated at the ωB97-XD/6–311++G(d,p), with the purpose to have a common base line for the comparison.

The $w_{2}^{SR}/w_{1}^{SR}$ computed ratio equals to 0.65 and 0.92 for the reaction occurring in the presence of Co^I^Cbx and Co^II^Cbx complexes, respectively, which in turn results in estimated values of $f_{R}G_{R}$ about 19.14 kcal/mol and 177.50 kcal/mol for each complex. It must be considered that, for both reactions, $G_{R}$ can be approximated as the difference between the ionization potential (IP) of either Co^I^Cbx or Co^II^Cbx, and the electron affinity (EA) of CH_3_Cl. Moreover, since the methyl halide is the same for both reactions, the difference in the $G_{R}$ term is solely defined by the IP values of Co^I^Cbx and Co^II^Cbx complexes, which are 47.33 kcal/mol and 173.44 kcal/mol, respectively. The latter is a very important result because it suggests that because of its lower IP value, the Co^I^Cbx complex performs as a better electron shuttle in comparison to Co^II^Cbx (see $\Delta E^{\neq }$ values from Table 1), an observation that has been previously reported by numerous biomimetic essays [37,62]. It is also important to point out that this result agrees with the reduction potential measured for different cobaloximes [62]. In the upcoming text, an analysis on the electron density within the QTAIM perspective will be presented to get insights on the changes occurring at RF critical points of both reactions. 

### 3.2. Source Function and QTAIM Analysis 

The topological analysis of the electron density at the IRC points associated with the different RF critical points, allows the localization and characterization of the BCPs of the atomic pairs (i.e., N–Co, Co–C, and C–Cl) that are relevant for the cleavage reactions mediated by the two cobalt complexes. At the extremes of the IRC (i.e., reactant and product states) of both reactions, the paths for the N–Co, Co–C, and C–Cl bonds form almost a straight line, except for the Co–C at $\xi_{R}$, and C–Cl at $\xi_{P}$, which show a curvature in the bond path near to the C and Cl attractors (see Figure S1). It is worth mentioning that this behavior is characteristic of non-covalent weak interactions [63]; thus, it can be suggested that the reactant as well as the product of the two reactions correspond to weakly bound non-covalent complexes. In a further step of the analysis, the BCP positions and the QTAIM atomic partition was used to compute the source function (SF). The SF was computed at the N–Co, Co–C, and C–Cl BCPs with the purpose of tracking the evolution of the interaction of these atomic pairs. As mentioned in the Models and Methods Section, the electron density at a given BCP (ρ(**r**_BCP_)) is not only determined by the contributions of the two interacting atoms, but also by all-remaining atoms of the system (see Equation (6)); thus, it is possible to include the “non-local” effects into the bonding analysis. Table 2 summarizes the computed ρ(**r**_BCP_), and S%(**r**_BCP_, Ω,Ω’) values for the methyl chloride dehalogenation mediated by Co^I^Cbx and Co^II^Cbx, respectively (additional information such as S(**r**_BCP_, Ω) is shown in Tables S1 and S2 of the Supporting Information). In the case of Co^II^Cbx, when the system evolves from $\xi_{R}$ to $\xi_{1}^{SR}$, a significant increase on the S(**r**_BCP_, Ω) as well as S%(**r**_BCP_, Ω_N_, Ω_Co_) values is observed, suggesting an increase in the N–Co interaction. No further changes in this atomic pair are evident in the other RF critical points. The opposite is observed in case of the reaction occurring on the Co^I^Cbx complex for which the changes on S(**r**_BCP_,Ω) and S%(**r**_BCP_, Ω_N_, Ω_Co_) indicate a continuous weakening of the N–Co covalent coordinated bond along the whole IRC. The latter represents an important result since it suggests that the lower axial ligand (i.e., 5,6-dimethylbenzimidazole) actively participates in the chemical process catalyzed by Co^I^. Regarding the other BCPs, located between Co–C and C–Cl pairs, the data in Table 2 indicate that only the former one has a dependence on the cobalt oxidation state. For the Co^II^Cbx system, the Co–C interaction is stronger than its counterpart at the $\xi_{TS}$ point as expected by considering the relatively short Co–C distance obtained for this case (see Table S3 in Supporting Information). For the Co^I^Cbx system, it is observed in Table 2 that the Co–C interaction increases rapidly from $\xi_{TS}$ to $\xi_{P}$, and it becomes higher than its Co^II^ counterpart at the state of the products. The latter result indicates that the Co–C bond is stronger as well as more stable in the CH_3_Co^I^Cbx products than in its CH_3_Co^II^Cbx counterpart. In agreement with the aforementioned, an inspection of Table 2 show that at $\xi_{P}$ S%(**r**_BCP_, Ω_Co_,Ω_C_) is 11.07% greater in the CH_3_Co^I^Cbx complex. Finally, for the C–Cl interaction, Table 2 shows a continuous weakening of the bond along the whole IRC, as suggested by the decrease in the of S%(**r**_BCP_, Ω_C_,Ω_Cl_) values. 

In a further stage of the study, the QTAIM charges were computed to better understand the electronic population associated with the atomic basins that are relevant to the reaction. Although these charges can be obtained for the whole IRC, emphasis is made at the IRC $\xi_{R}$, $\xi_{TS}$, and $\xi_{P}$ points. The computed charges are summarized in Table 3, where it is observed that at the reactants state, the Co^I^Cbx complex has a net charge (q(Ω)) of nearly −1, in agreement with an oxidation state of 1+ in the Co atom; whereas the Co^II^Cbx system has a charge close to 0, according to an oxidation state 2+ associated with the Co atom. As the reaction advances from $\xi_{R}$ to $\xi_{P}$, the charge of the nucleophile of both reactions increases significantly indicating the oxidation of the cobalt atom regardless of its initial electron count. In order to determine the destination of the electron that is being released by the cobalt atom, the charges of the CH_3_ fragment as well as the Cl atom must be considered. In agreement with our results previously reported in Reference. [35], the charge of the Cl leaving group constantly decreases as the reaction advances on the presence of the Co^I^Cbx complex, suggesting a heterolytic C–Cl cleavage stabilized by the solvent medium and the consequent formation of a stable Co–CH_3_ covalently coordinated bond. Although a homolytic C–Cl cleavage would be expected in the reaction mediated by the Co^II^Cbx complex [36], the data in Table 3 indicates that the Cl leaving atom presents the exact same behavior reported for the reaction occurring on the Co^I^Cbx complex. This relevant result means that an unusual two-center one-electron bond is being formed between the cobalt and the carbon atoms of the CH_3_Co^II^Cbx product (see Scheme 1). It is important to point out that, although uncommon, these kinds of bonds are not rare, and they play an important role in the chemistry of radicals [64,65,66,67,68]. 

### 3.3. KLD Analysis

**Figure 3:**
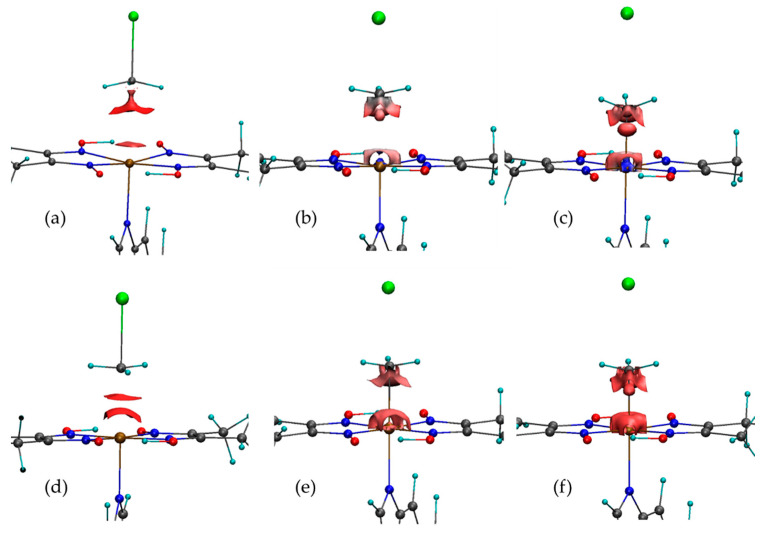
Representation of the $\chi_{XC}^{\sigma }$ computed at (**a**) $\xi_{TS}^{}$ (isovalue = 0.200 bte), (**b**) $\xi_{1}^{SP}$ (isovalue = 0.341 bte), (**c**) $\xi_{P}^{}$ (isovalue 0.375 bte) for the reductive de halogenation mediated by Co^I^Cbx and at (**d**) $\xi_{TS}^{}$ (isovalue = 0.340 bte), (**e**) $\xi_{3}^{SP}$ (isovalue = 0.302 bte), (**f**) $\xi_{P}^{}$ (isovalue = 0.340 bte) for the reaction mediated by Co^II^Cbx. C atoms are in gray, with Co in ochre, N in blue, O in red and Cl in green.

Analysis of the electron localization obtained by means of the KLD method was employed as a tool to further characterize the two-center one-electron bond in CH_3_Co^II^Cbx product and compare it with the results obtained for its CH_3_Co^I^Cbx counterpart. To reduce the computational cost, the $\chi_{XC}^{\sigma }$ was obtained in a box with volume = 16 Å^3^ located around the Co–C BCP. For both, Co^I^Cbx and Co^II^Cbx cases, appreciable electron localization basins are not observed in the computed box at $\xi_{TS}$, and only fractions of the C–H basins and the Co core electrons (Figure 3a,d) were identified, even though, in the Co^II^Cbx case, the Co–C distance is 0.61 Å shorter than in the Co^I^Cbx case (see Table S3). At $\xi_{1}^{SP}$, in the reaction mediated by Co^I^Cbx, $\chi_{XC}^{\sigma }$ reveals a well-defined bond basin in the interatomic Co–C region with an isovalue of 0.341 bte (Figure 3b). On the other hand, a defined bond basin is absent at $\xi_{1}^{SP}$ for the reaction mediated by Co^II^Cbx, and only some electron localization can be detected when a low isovalue of 0.218 bte (Figure 3e) is considered. As depicted in Figure 3c,f, for both cases, Co^I^Cbx and Co^II^Cbx, Co–C bond basins are observed at $\xi_{P}$. In the former case, the bond basin appears at $\chi_{XC}^{\sigma }$ = 0.385 bte and integrates to 0.604 electrons, while in the latter case, the bond basin with 0.299 electrons appears at $\chi_{XC}^{\sigma }$ = 0.345 bte. The latter results indicate that the two-center two-electron bond of the CH_3_Co^I^Cbx possesses a higher degree of localization and accounts for a larger number of electrons; thus, it is possible to suggest that the Co–C bond in the CH_3_Co^I^Cbx system is well-defined and more stable than its counterpart present in the CH_3_Co^II^Cbx product.

**Figure 4:**
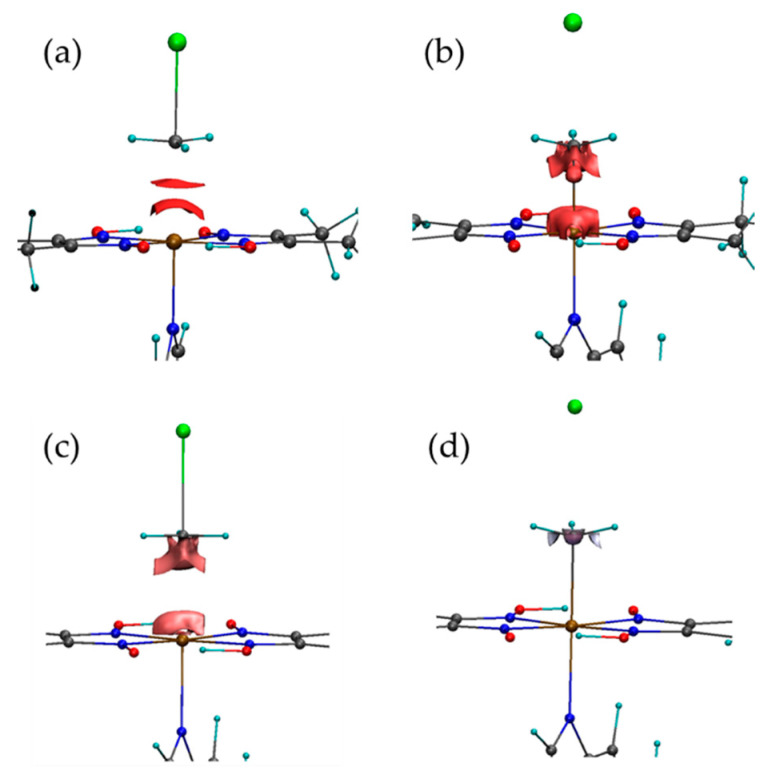
(**a**) Showing $\chi_{XC}^{\alpha }$ for the C–Cl cleavage mediated by Co^II^Cbx at $\xi_{TS}$ (isovalue = 0.220 bte) and (**b**) $\xi_{P}$ (isovalue =0.345 bte), respectively. (**c**) $\chi_{XC}^{\beta }$ for the C–Cl cleavage mediated by Co^II^Cbx at $\xi_{TS}$ (isovalue = 0.220 bte) and (**d**) $\xi_{P}$ (isovalue =0.345 bte), respectively. C atoms are in gray, with Co in ochre, N in blue, O in red and Cl in green.

**Figure 5:**
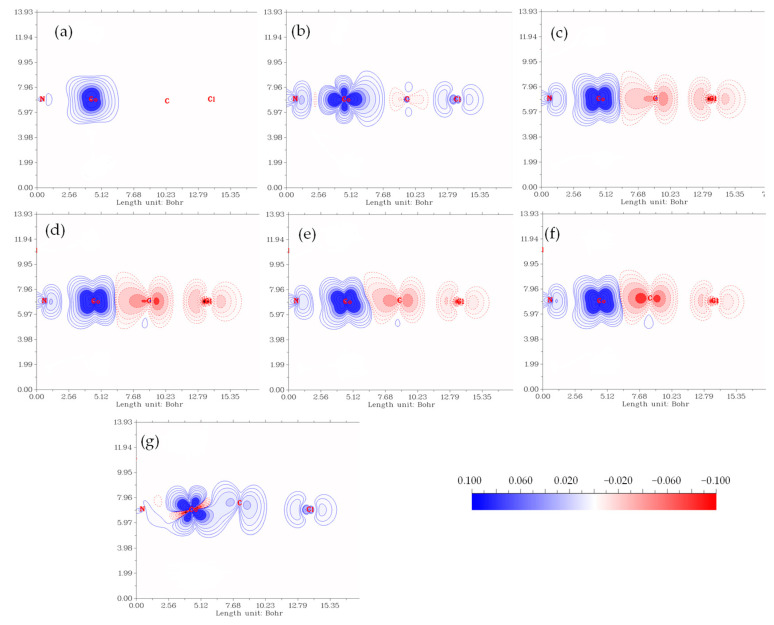
Electron spin-density contours maps of the reaction mediated by the Co^II^Cbx at the IRC points (**a**) $\xi_{R}$, (**b**) $\xi_{1}^{SR}$, (**c**) $\xi_{TS}$, (**d**) $\xi_{1}^{SP}$, (**e**) $\xi_{2}^{SP}$, (**f**) $\xi_{3}^{SP}$, and (**g**) $\xi_{P}$. All the maps are parallel to the Co–C interaction, and perpendicular to the basal plane of the Co^II^Cbx complex. The color scale is general for all the graphs.

An advantage of our electron localization method is that it allows the independent analysis of α and β electronic states when studying open-shell systems, as required in the present case (i.e., CH_3_Co^II^Cbx). Isosurfaces of the $\chi_{XC}^{\alpha }$ function, at $\xi_{TS}$ and $\xi_{P}$, are shown in Figure 4a,b, while plots of its counterpart $\chi_{XC}^{\beta }$ are shown in Figure 4c,d. Examination of these Figures allows one to conclude that the Co–C bond in the product of the reaction mediated by Co^II^Cbx is solely defined by α-electrons. These results agree with the spin population (Table 3, last column) and spin density plots (Figure 5). According to the spin population and spin electron density, at $\xi_{R}$ the unpaired electron is mainly concentrated over the Co^II^Cbx fragment, particularly over the cobalt atom (Figure 5a), whereas at $\xi_{P}$, an important α-spin density concentration is observed at the cobalt (78%) and carbon (18%) atoms (Figure 5g). At the intermediate points (Figure 5b–f and Table 3) α-states electron are mainly present on the Co atom, whereas β-states are observed principally in the C–Cl bond. Accordingly, as the C–Cl breaks, the Co atom transfers some β-spin electrons to the C atom, and at the same time, the C atom transfers some α-spin electrons to the Co atom. In summary, at the beginning of the chemical process, the unpaired electrons are almost exclusively located at the Co atom, while at the end of the reaction, these electrons are shared between the Co and the C; however, certain preference of the electrons to occupy the α-states is determined. Finally, the results depicted in Figure 5c indicate that the Co^II^Cbx and CH_3_Cl fragments are unsymmetrically polarized at the $\xi_{TS}$ point of the IRC. The latter result partly explains the larger instability of the Co^II^Cbx–CH_3_– Cl transition state in comparison to its counterpart containing Co^I^.

## 4. Conclusions

The effect of the cobalt oxidation state in the C–X bond cleavage of methyl halides mediated by Co[N]Cbx was theoretically investigated in the present work. For this purpose, several analysis tools were employed to track changes in the reacting system along the IRC. In first place, the RF analysis provided a reliable description of the reaction, acting as a tool for identifying the points in the IRC where the most relevant changes occur. Moreover, the $\Delta E^{\neq }$ destabilizing term ($f_{R}G_{R}$), was estimated through the RF formalism, showing that the Co^I^Cbx performs as a better electron shuttle than its Co^II^Cbx counterpart. In a further step, the SF analysis shows that the axial ligand, 5,6-dimethylbenzimidazole, has a significant role only in the reaction mediated by Co^I^Cbx throughout the whole IRC. Furthermore, the SF and the QTAIM charges indicate that the C–Cl bond cleavage occurs heterolytically, regardless of the oxidation state of the cobalt atom. Accordingly, the product obtained from the reaction mediated by Co^II^Cbx is characterized by the formation of an uncommon two-center one-electron bond between Co and C atoms. The latter was further confirmed through the $\chi_{XC}^{\sigma }$ descriptor, which provides a pictorial description of the localization of α-electrons in the Co–C interatomic region. Finally, the existence of this two-center one-electron bond explains the endothermic character reaction mediated by Co^II^Cbx.

## Figures and Tables

**Table 1:** RF works, activation ($\Delta E^{\neq }$), and reaction (∆E^0^) energies in kcal/mol, calculated at the uωB97-XD/6–311++G(d,p) level and obtained from the IRC profiles.

Methyl Halide	$w_{1}^{SR}$	$w_{2}^{SR}$	$w_{1}^{SP}$	$w_{2}^{SP}$	$w_{3}^{SP}$	$w_{4}^{SP}$	ΔE^≠^	ΔE^0^
CH_3_Cl	11.1 (3.5)	10.2 (2.3)	−0.3 (−19.0)	−0.4 (−29.4)	−1.8	−2.1	21.3 (5.7)	16.5 (−42.7)
CH_3_Br	11.6 (3.3)	9.5 (1.7)	−0.6 (−16.8)	−2.7 (−27.2)	---	---	21.1 (5.0)	17.8 (−39.1)
CH_3_I *	9.4 (2.3)	6.7 (0.6)	−0.4 (−21.6)	−0.7 (−31.6)	−2.4	−3.3	16.0 (2.8)	9.1 (−50.4)

* The 6-311(d,p) basis set was adopted for the I atom. In parenthesis, works and energies are shown for the reactions mediated by Co^I^Cbx calculated at ωB97-XD/6–311++G(d,p) level.

**Table 2:** Source function description at the N–Co, Co–C, and C–Cl BCPs interactions measured in the IRC points $\xi_{R}$, $\xi_{1}^{SR}$, $\xi_{TS}$, $\xi_{1}^{SP}$, and $\xi_{P}$ for the dehalogenation of chloromethane mediated by Co^I^Cbx.

Interaction (A–B)	IRC	ρ(r_BCP_) ^1^		S%(r_bcp_,Ω_A_,Ω_B_)	
		Co^I^Cbx	Co^II^Cbx	Co^I^Cbx	Co^II^Cbx
N–Co	$\xi_{R}$	1.00 × 10^−1^	7.94 × 10^−2^	60.03	25.97
	$\xi_{1}^{SR}$	9.53 × 10^−2^	7.28 × 10^−2^	57.72	46.45
	$\xi_{TS}$	9.22 × 10^−2^	7.69 × 10^−2^	56.27	47.96
	$\xi_{1}^{SP}$	8.42 × 10^−2^	7.77 × 10^−2^	51.73	47.57
	$\xi_{P}$	7.25 × 10^−2^	7.63 × 10^−2^	42.61	46.38
Co–C	$\xi_{R}$	1.07 × 10^−2^	8.41 × 10^−3^	−169.05	−213.8
	$\xi_{1}^{SR}$	1.94 × 10^−2^	1.85 × 10^−2^	−84.41	−98.81
	$\xi_{TS}$	2.71 × 10^−2^	4.84 × 10^−2^	−40.43	−27.27
	$\xi_{1}^{SP}$	5.94 × 10^−2^	5.44 × 10^−2^	28.60	21.34
	$\xi_{P}$	1.25 × 10^−1^	9.44 × 10^−2^	61.16	50.09
C–Cl	$\xi_{R}$	1.64 × 10^−1^	1.69 × 10^−1^	82.69	83.65
	$\xi_{1}^{SR}$	1.24 × 10^−1^	1.03 × 10^−1^	77.89	75.14
	$\xi_{TS}$	8.78 × 10^−2^	4.92 × 10^−2^	69.48	48.61
	$\xi_{1}^{SP}$	3.66 × 10^−2^	4.27 × 10^−2^	15.73	40.25
	$\xi_{P}$	9.99 × 10^−3^	1.66 × 10^−2^	−48.95	−13.02

^1^ All the values are given in e a_0_^−3^.

**Table 3:** Bader charges, for reactions mediated by Co^I^Cbx and Co^II^Cbx in the indicated IRC points. For reaction mediated by Co^II^Cbx explicit spin α and β charges and spin population are shown.

		Co^I^Cbx	Co^II^Cbx			
IRC Point	Ω	q (Ω) (e)	q (Ω) (e)	q^α^ (Ω) (e)	q^β^ (Ω) (e)	Nspin ^2^
$\xi_{R}$	Nu ^1^	−0.946	+0.014	+113.007	+114.008	1.00
	CH_3_	+0.245	+0.260	+4.631	+4.654	0.02
	Cl	−0.304	−0.275	+8.362	+8.337	−0.02
$\xi_{1}^{SR}$	Nu ^1^	−0.868	+0.028	+113.034	+113.993	0.96
	CH_3_	+0.276	+0.329	+4.684	+4.675	−0.01
	Cl	−0.409	−0.357	+8.282	+8.332	0.05
$\xi_{TS}$	Nu ^1^	−0.711	+0.395	+113.065	+114.342	1.28
	CH_3_	+0.269	+0.294	+4.740	+4.553	−0.19
	Cl	−0.558	−0.689	+8.195	+8.105	−0.09
$\xi_{1}^{SP}$	Nu ^1^	−0.215	+0.498	+113.067	+114.440	1.37
	CH_3_	+0.197	+0.253	+4.760	+4.486	−0.27
	Cl	−0.979	−0.750	+8.173	+8.075	−0.10
$\xi_{2}^{SP}$	Nu ^1^	---	+0.495	+113.119	+114.383	1.26
	CH_3_	---	+0.274	+4.742	+4.527	−0.21
	Cl	---	−0.769	+8.141	+8.090	−0.05
$\xi_{3}^{SP}$	Nu ^1^	---	+0.687	+113.152	+114.556	1.40
	CH_3_	---	+0.177	+4.756	+4.407	−0.35
	Cl	---	−0.865	+8.092	+8.037	−0.05
$\xi_{P}$	Nu ^1^	+0.170	+0.712	+113.471	+114.247	0.78
	CH_3_	−0.194	+0.181	+4.500	+4.678	0.18
	Cl	−0.976	−0.893	+8.029	+8.074	0.04

^1^ Nu refers to either the Co^I^Cbx or Co^II^Cbx fragments. ^2^ Nspin = Nα(Ω)-Nβ(Ω).

## Data Availability

Not applicable.

## Supplementary Materials

The following supporting information can be downloaded at: https://www.mdpi.com/article/10.3390/molecules27217283/s1, Figure S1: Localization of the critical points of ρ for the five IRC points indicated for the reaction mediated by CoIICbx. Table S1. Atomic distances D_Co–C_ and D_C–Cl_ for reactions mediated by Co^I^Cbx and Co^II^Cbx, at the IRC points indicated. Table S2. Source function description at the N–Co, Co–C, and C–Cl BCPs interactions measured at the IRC points $\xi_{R}$, $\xi_{1}^{SR}$, $\xi_{TS}$, $\xi_{1}^{SP}$, $\xi_{2}^{SP}$, $\xi_{3}^{SP}$, and $\xi_{P}$ for the dehalogenation of chloromethane mediated by Cob^II^Cbx. Table S3. Source function description at the N–Co, Co–C, and C–Cl BCPs interactions measured in the IRC points $\xi_{R}$, $\xi_{1}^{SR}$, $\xi_{TS}$, $\xi_{1}^{SP}$, and $\xi_{P}$ for the dehalogenation of chloromethane mediated by CoICbx.

## Sample Availability

Samples of the compounds are not available from the authors.
