# Supplementary material for: A Theoretical Study of the C–X Bond Cleavage Mediated by Cob(II)Aloxime
Source: Molecules. 2022 Oct 26;27(21):7283. doi: 10.3390/molecules27217283 (PMC9658964; doi:10.3390/molecules27217283)
Supplement: Supplementary file 1 [file molecules-27-07283-s001.zip › molecules-1956573-supplementary.pdf]

Supporting Information

A theoretical study of the C–X bond cleavage mediated by Cob(II)aloxime

Luis E. Seijas[1], Cesar H. Zambrano[2], Vladimir Rodríguez[3], Jorge Alí-Torres[4], Luis Rincón[2], and F. Javier Torres[1,2*],

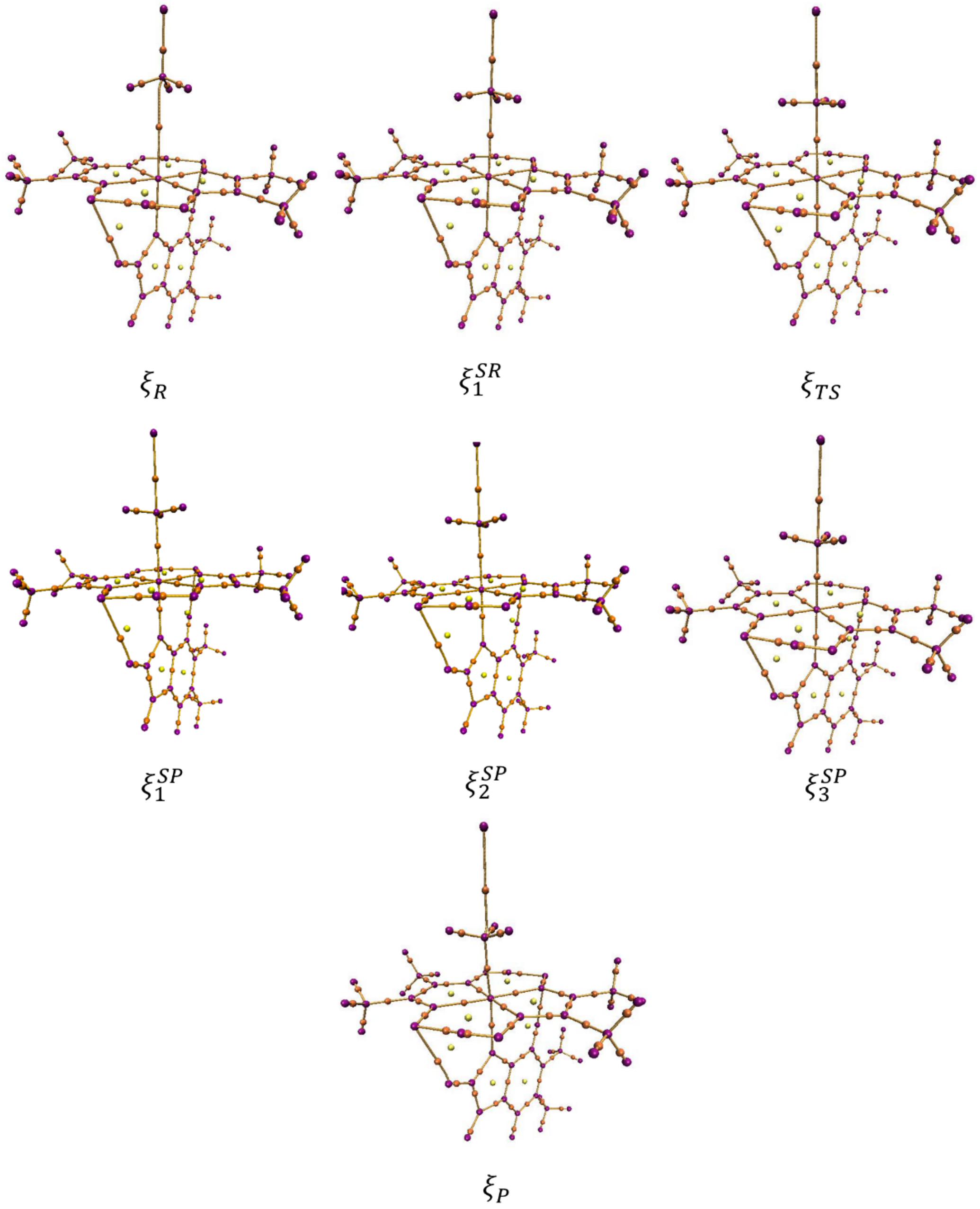

Figure S1: Localization of the critical points of ρ for the five IRC points indicated for the reaction mediated by CoIICbx.

Table S1. Source function description at the N–Co, Co–C, and C–Cl BCPs interactions measured at the IRC points ξ_R , ξ_1^{SR}, ξ_{TS}, ξ_1^{SP}, ξ_2^{SP}, ξ_3^{SP}, and ξ_P , for the dehalogenation of chloromethane mediated by $Cob^{II}Cbx$.

IRC	Interaction	$\rho(r_{BCP})$	$S(r_{bcp},N)$ [1]	$S(r_{bcp},Co)$ [1]	$S(r_{bcp},C)$ [1]	$S(r_{bcp},Cl)$ [1]	$S\%(r_{bcp},N+Co)$	$S\%(r_{bcp},Co+C)$	$S\%(r_{bcp},C+Cl)$
ξ_R	N–Co	7.94×10^{-02}	1.77×10^{-03}	1.90×10^{-02}	---	---	25.97	---	---
	Co–C	8.41×10^{-03}	---	-7.82×10^{-03}	-1.02×10^{-02}	---	---	−213.8	---
	C–Cl	1.69×10^{-01}	---	---	5.77×10^{-02}	8.46×10^{-02}	---	---	83.65
ξ_1^{SR}	N–Co	7.28×10^{-02}	1.76×10^{-02}	1.68×10^{-02}	---	---	46.45	---	---
	Co–C	1.85×10^{-02}	---	-8.12×10^{-03}	-1.01×10^{-02}	---	---	−98.81	---
	C–Cl	1.03×10^{-01}	---	---	2.62×10^{-02}	5.10×10^{-02}	---	---	75.14
ξ_{TS}	N–Co	7.69×10^{-02}	2.03×10^{-02}	1.69×10^{-02}	---	---	47.96	---	---
	Co–C	4.84×10^{-02}	---	-3.41×10^{-03}	-4.14×10^{-03}	---	---	−27.27	---
	C–Cl	4.92×10^{-02}	---	---	6.52×10^{-04}	2.29×10^{-02}	---	---	48.61
ξ_1^{SP}	N–Co	7.77×10^{-02}	2.06×10^{-02}	1.60×10^{-02}	---	---	47.57	---	---
	Co–C	5.44×10^{-02}	---	7.04×10^{-03}	4.59×10^{-03}	---	---	21.34	---
	C–Cl	4.27×10^{-02}	---	---	-2.51×10^{-03}	1.97×10^{-02}	---	---	40.25
ξ_2^{SP}	N–Co	7.62×10^{-02}	2.00×10^{-02}	1.58×10^{-02}	---	---	47.06	---	---
	Co–C	5.90×10^{-02}	---	8.98×10^{-03}	6.84×10^{-03}	---	---	26.79	---
	C–Cl	3.60×10^{-02}	---	---	-4.60×10^{-03}	1.68×10^{-02}	---	---	33.95
ξ_3^{SP}	N–Co	7.56×10^{-02}	1.98×10^{-02}	1.59×10^{-02}	---	---	46.65	---	---
	Co–C	8.07×10^{-02}	---	9.08×10^{-03}	7.69×10^{-03}	---	---	37.06	---
	C–Cl	2.57×10^{-02}	---	---	-8.80×10^{-03}	1.04×10^{-02}	---	---	12.47
ξ_P	N–Co	7.63×10^{-02}	2.02×10^{-02}	1.59×10^{-02}	---	---	46.38	---	---
	Co–C	9.44×10^{-02}	---	2.34×10^{-02}	2.40×10^{-02}	---	---	50.09	---
	C–Cl	1.66×10^{-02}	---	---	-9.69×10^{-03}	7.59×10^{-03}	---	---	−13.02

[1] All the values are given in e a_0^{-3}.

Table S2. Source function description at the N–Co, Co–C, and C–Cl BCPs interactions measured in the IRC points ξ_R , ξ_1^{SR}, ξ_{TS}, ξ_1^{SP}, and ξ_P for the dehalogenation of chloromethane mediated by Co^{I}Cbx.

IRC	Interaction	ρ(r_{BCP})	S(r_{BCP},N) [1]	S(r_{BCP},Co) [1]	S(r_{BCP},C) [1]	S(r_{BCP},Cl) [1]	S%(r_{bcp},N+Co)	S%(r_{bcp},Co+C)	S%(r_{bcp},C+Cl)
	N–Co	1.00×10^{-01}	3.17×10^{-02}	3.00×10^{-02}	---	---	60.03	---	---
ξ_R	Co–C	1.07×10^{-02}	---	-7.71×10^{-03}	-1.04×10^{-02}	---	---	−169.05	---
	C–Cl	1.64×10^{-01}	---	---	5.40×10^{-02}	8.18×10^{-02}	---	---	82.69
	N–Co	9.53×10^{-02}	2.89×10^{-02}	2.72×10^{-02}	---	---	57.72	---	---
ξ_1^{SR}	Co–C	1.94×10^{-02}	---	-6.25×10^{-03}	-1.01×10^{-02}	---	---	−84.41	---
	C–Cl	1.24×10^{-01}	---	---	3.50×10^{-02}	6.16×10^{-02}	---	---	77.89
	N–Co	9.22×10^{-02}	2.59×10^{-02}	2.71×10^{-02}	---	---	56.27		---
ξ_{TS}	Co–C	2.71×10^{-02}	---	-3.57×10^{-03}	-7.43×10^{-03}	---	---	−40.43	---
	C–Cl	8.78×10^{-02}	---		1.80×10^{-02}	4.28×10^{-02}	---	---	69.48
	N–Co	8.42×10^{-02}	2.30×10^{-02}	2.15×10^{-02}	---	---	51.73	---	---
ξ_1^{SP}	Co–C	5.94×10^{-02}	---	9.01×10^{-03}	8.02×10^{-03}	---	---	28.60	---
	C–Cl	3.66×10^{-02}	---		-6.55×10^{-03}	1.67×10^{-02}	---	---	15.73
	N–Co	7.25×10^{-02}	1.79×10^{-02}	1.39×10^{-02}	---	---	42.61	---	---
ξ_P	Co–C	1.25×10^{-01}	---	3.88×10^{-02}	3.81×10^{-02}	---	---	61.16	---
	C–Cl	9.99×10^{-03}	---	---	-3.41×10^{-03}	6.86×10^{-03}	---	---	−48.95

[1] All the values are given in e a_0^{-3}.

Table S3. Atomic distances $D_{Co–C}$ and $D_{C–Cl}$ for reactions mediated by Co^{I}Cbx and Co^{II}Cbx, at the IRC points indicated.

	**D**	ξ_R	ξ_1^{SR}	ξ_{TS}	ξ_1^{SP}	ξ_2^{SP}	ξ_3^{SP}	ξ_P
Co^{I}Cbx	Co–C	3.20	2.84	2.71	2.36	--	--	1.95
	C–Cl	1.82	1.95	2.10	2.48	--	--	3.05
Co^{II}Cbx	Co–C	3.19	2.64	2.38	2.33	2.27	2.14	2.05
	C–Cl	1.81	2.06	2.37	2.43	2.50	2.66	2.98
